# A fluorescent bimolecular complementation screen reveals MAF1, RNF7 and SETD3 as PCNA-associated proteins in human cells

**DOI:** 10.1080/15384101.2015.1053667

**Published:** 2015-06-01

**Authors:** Simon E Cooper, Elsie Hodimont, Catherine M Green

**Affiliations:** 1 Department of Zoology; University of Cambridge; Cambridge, UK; 2 Wellcome Trust Center for Human Genetics; University of Oxford; Oxford, UK

**Keywords:** Bimolecular Fluorescence Complementation, DNA Replication, Maf1, PCNA, Protein interactions, RNF7, SetD3

## Abstract

The proliferating cell nuclear antigen (PCNA) is a conserved component of DNA replication factories, and interactions with PCNA mediate the recruitment of many essential DNA replication enzymes to these sites of DNA synthesis. A complete description of the structure and composition of these factories remains elusive, and a better knowledge of them will improve our understanding of how the maintenance of genome and epigenetic stability is achieved. To fully characterize the set of proteins that interact with PCNA we developed a bimolecular fluorescence complementation (BiFC) screen for PCNA-interactors in human cells. This 2-hybrid type screen for interactors from a human cDNA library is rapid and efficient. The fluorescent read-out for protein interaction enables facile selection of interacting clones, and we combined this with next generation sequencing to identify the cDNAs encoding the interacting proteins. This method was able to reproducibly identify previously characterized PCNA-interactors but importantly also identified RNF7, Maf1 and SetD3 as PCNA-interacting proteins. We validated these interactions by co-immunoprecipitation from human cell extracts and by interaction analyses using recombinant proteins. These results show that the BiFC screen is a valuable method for the identification of protein-protein interactions in living mammalian cells. This approach has potentially wide application as it is high throughput and readily automated. We suggest that, given this interaction with PCNA, Maf1, RNF7, and SetD3 are potentially involved in DNA replication, DNA repair, or associated processes.

## Introduction

The process of chromosomal replication is a complex one. Not only must the DNA sequence be completely copied, and any mistakes produced during the copying process accurately rectified, but the associated chromatin structures must also be properly reproduced to generate 2 daughter cells containing the same information as the parent cell. Failures in these copying processes result in the inheritance of altered genetic and epigenetic information, which can lead to cell death, or the development of cancer. While the enzymatic requirements for the accurate copying of genetic information are well understood, the mechanisms that exist to control and coordinate the other events of chromosomal replication are less well defined.^1^

Proliferating cell nuclear antigen (PCNA) is a conserved DNA sliding clamp protein essential for DNA replication in eukaryotic cells. This small (29kDa monomer size in humans) protein forms a homotrimeric toroidal structure which encircles the DNA at the replication fork. Its capacity to translocate over the newly synthesized duplex means that it can act as a sliding recruitment protein; proteins which bind to PCNA are brought into the vicinity of the active replication fork and the nascent DNA strands. Thus, although it possesses no enzymatic activity, PCNA is used to concentrate the enzymes of DNA replication at their sites of action and modulate their activity.^2-4^

Many proteins have been convincingly shown to interact with PCNA. These include the DNA polymerases epsilon and delta (PolE and PolD), required for DNA synthesis;^5-8^ flap endonuclease 1 (Fen1) and ligase I (Lig1) for Okazaki fragment processing^9,10^; Mut S homologs 3 (MSH3) and 6 (MSH6) for mismatch repair^11,12^; chromatin assembly factor 1 (Caf-1)^13^; and DNA methyltransferase 1 (DNMT1) for epigenetic inheritance,^14^ among many others. Thus PCNA is central to many of the processes that must occur in a coordinated way as chromosomal replication proceeds.^15^ PCNA also interacts with additional partners involved in more specialized pathways. As examples, PCNA also regulates translesion DNA synthesis via interaction with polymerase eta (PolH)^16-18^; cell cycle arrest via p21^19-21^; S-phase specific protein degradation via the Cullin 4 (Cul4)/DNA damage binding protein 1 (DDB1) ubiquitin ligase,^22-24^ nucleotide excision repair via xeroderma pigmentosum proteins A and G (XPA/XPG),^25,26^ and base excision repair via apurinic/apyrimidinic endonuclease 1 (Ape1),^27^ uracil DNA glycosylase (UNG2)^28^ and 3-methyladenine-DNA glycosylase (MPG)^29^; and this list is by no means exhaustive.

For the most part, interactions between PCNA and its protein partners have been identified on a case-by-case basis. Many interactions were initially identified by immunoprecipitation or *in vitro* binding of recombinant proteins, and it was subsequently noticed that PCNA-interacting proteins often contain the PCNA interacting protein (PIP) motif.^30,31^ This is a small peptide motif with consensus sequence Q-x-x-[LIM]-x-x-[FY]-[FY], derivatives of which are found in PolD3, Fen1, Lig1, MSH3, MSH6, Caf-1, DNMT1, PolH, p21, XPG, Ape1, UNG2 and MPG. Crystal structures of PCNA with interacting proteins or peptides have demonstrated that this motif is a direct binding surface, interacting with the inter-domain connecting loop of PCNA in a mainly hydrophobic manner.^32^ Proteins which contain such a motif on a solvent-exposed surface are therefore good candidates for PCNA interactors. However, there are some characterized PCNA-interacting proteins that do not contain such a motif. As examples, the catalytic subunit of PolD likely binds PCNA via a “KA-box”,^33^ and the NER protein XPA uses a so-called APIM (AlkB homolog 2 PCNA interacting motif) for its PCNA interaction.^34^ All these motifs are degenerate and short, thus a bioinformatics-based search of the human proteome is unlikely to identify specifically all the true PCNA-interactors. Given the importance of PCNA in regulating the processes that ensure genome and epigenome stability through replication, it has been previously noted that a full characterization of the PCNA-interactome would be desirable.^4,35^

**Figure 1.**
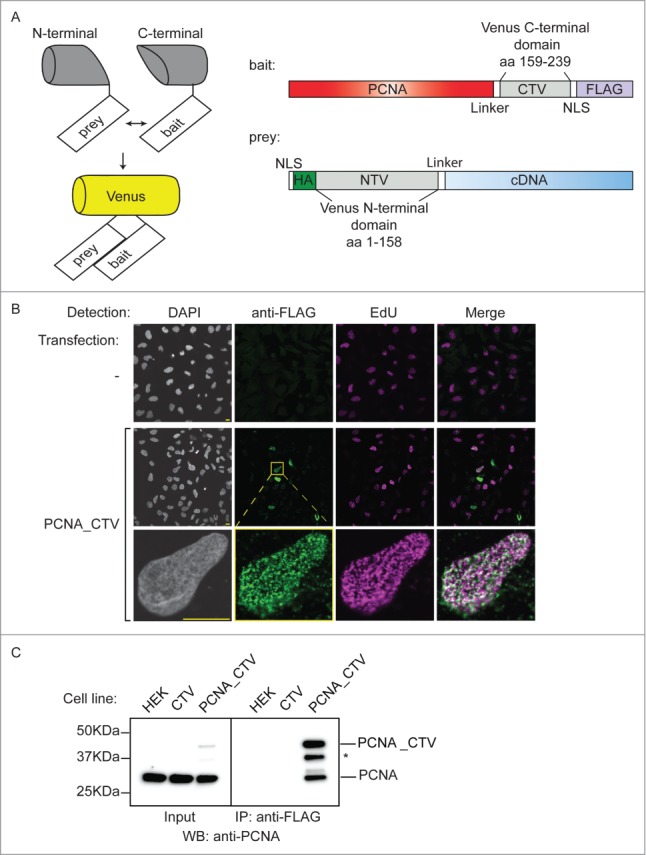
Production of a bait cell line for the BiFC screen. (**A**) Schematic of the BiFC principle and constructs used. The C-terminal and N-terminal portions of Venus fluorescent protein are individually non-fluorescent but they fold to a fluorescent state when brought into proximity by the interaction of bait and prey proteins. The bait construct is based on pcDNA3.1 and produces PCNA protein fused to a linker, the C-terminal 80 amino acids of Venus, a nuclear localization signal (NLS) and a FLAG epitope. The prey construct is based on the episomally maintained pCEP4 plasmid and produces the product of the library cDNA with an NLS, an HA epitope, the first 158 amino acids of Venus and a linker region fused at its N-terminus. (**B**) The PCNA bait construct localizes to replication factories. MRC5 cells were transfected as indicated and then processed for immunofluorescence and EdU detection after a 10 minute EdU incorporation and triton extraction prior to fixation. EdU is incorporated at sites of DNA synthesis and was visualised using a click reaction and Alexa Fluor 647 azide (magenta). An anti-FLAG mouse monoclonal antibody was used with an anti-mouse IgG Alexa Fluor 488 secondary antibody to visualize the bait protein (green). Images were acquired using confocal microscopy at excitation wavelengths: 405 nm (DAPI nuclear stain); 488 nm (bait), 633 nm (DNA replication foci). Bar = 10µm. (**C**) The PCNA bait interacts with endogenous PCNA. Soluble nuclease-treated protein extracts were prepared from HEK293 cells or derivatives of (CTV and PCNA_CTV). PCNA_CTV was immuno-precipitated using the anti-FLAG monoclonal antibody and precipitates and input extracts analyzed by western blotting using anti-PCNA antibody. *non-specific band or degradation product.

We developed an in-cell screening approach to identify PCNA interaction partners. The format of our screen will allow it to report on interactions that happen in the context of active replication sites, even those that are DNA-dependent or transient, interactions that could be missed in a purification-based strategy. We based our screen on bimolecular fluorescence complementation (BiFC), the process whereby 2 fragments of a fluorescent protein, individually non-fluorescent, can combine to give a fluorescent species when brought into close proximity by the interaction of “bait” and “prey” proteins (**Fig. 1A**).^36,37^ A similar system has previously been used in the identification of proteins that interact with the protein kinase PKB/Akt.^38^ Here, we combined this strategy with fluorescent activated cell sorting (FACS) and next generation sequencing to develop a novel format for screening for protein interactions in real time in living mammalian cells.

## Results

To identify novel PCNA interacting proteins in human cells we used a bimolecular complementation (BiFC) approach with a PCNA “bait” (**Fig. 1A**). This comprises the full length PCNA open reading frame with the C-terminal portion of Venus fluorescent protein^39^ (CTV: amino acids 159–238) fused to its C-terminus. The split point of Venus was selected at between amino acids 158 and 159 after Remy *et al.*^*38*^ The construct also contains a linker sequence to minimise potential perturbations to PCNA folding,^40^ a nuclear localization signal (NLS) to ensure appropriate cellular location and a FLAG epitope for detection. Using indirect immunofluorescence after transient transfection in MRC5 cells we showed that the PCNA_CTV is recruited to focal sites of DNA replication (so-called replication factories^41,42^) where it co-localized with EdU incorporation in a triton-resistant manner (**Fig. 1B**). This suggests that the bait construct is loaded onto chromatin in a manner reminiscent of endogenous PCNA demonstrating that this tagged version of PCNA can recapitulate the essential cellular activity of PCNA.

We generated a cell line derived from HEK293 cells that stably expresses this construct from the CMV promoter. Western blotting of total cell extracts from control and bait cells using a polyclonal antibody against PCNA showed that the construct is expressed at levels well below that of endogenous PCNA (**Fig. 1C** - inputs), and immunoprecipitation of the PCNA_CTV from cell extracts using the anti-FLAG monoclonal antibody co-precipitated endogenous PCNA (**Fig. 1C**). Thus, the tagged bait is able to associate normally with endogenous PCNA to form mixed trimers, implying that its function is unlikely to be dramatically impaired.

**Figure 2.**
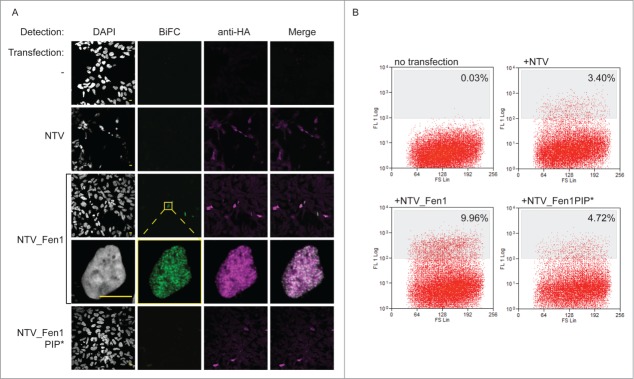
The BiFC approach reports on physiologically relevant protein interactions. (**A**) BiFC visualized by confocal microscopy. HEK293 cells stably expressing PCNA_CTV were transfected as indicated. Cells were processed for indirect immunofluorescence using a mouse anti-HA monoclonal primary antibody and goat anti-mouse Alexa Fluor 633 secondary antibody to detect the prey constructs (magenta) and BiFC signal was detected in the yellow channel, pseudo colored here in green. Bar = 10 µm. BiFC signal is specifically detected when the bait and prey constructs are able to interact. (**B**) BiFC detected by flow cytometry. PCNA_CTV expressing cells were transfected as indicated and yellow fluorescence monitored after 24 hours on a MoFlo cytometer. The percentage of cells above a threshold of 108 fluorescent units is indicated.

To validate and optimise the BiFC screen parameters we constructed positive and negative control “prey” constructs based on pCEP4, an episomally maintained plasmid. They express the target prey with the N-terminal part of Venus (NTV: amino acids 1–158) fused to the N-terminus (**Fig. 1A**). The constructs also contain an NLS, linker sequence and the HA epitope. Expression is driven by the CMV promoter and selection is by hygromycin resistance. As a positive control we fused the N-terminal part of Venus to the Fen1 open reading frame. The interaction between Fen1 and PCNA is well documented, occurs at replication factories and is mediated by the PIP box motif of Fen1.^9,43^ Mutation of the Fen1 PIP-box abolishes its interaction with PCNA^44,45^ so a mutated negative control version (L340A, F343A, F344A: PIP*) was also generated. We transiently transfected PCNA_CTV (bait) expressing cells with these NTV_Fen1 constructs. The wild type (WT) NTV_Fen1 transfection generated yellow fluorescent signal as a result of bimolecular fluorescence complementation (BiFC) mediated by the interaction between PCNA_CTV and NTV_Fen1 (**Fig. 2A**). Importantly, far less yellow fluorescence was detected in cells transfected with either the empty prey vector, or the mutated NTV_Fen1 PIP* construct that is unable to interact with PCNA (**Fig. 2A**). This shows that the BiFC signal is a sensitive and accurate reporter of biologically relevant protein associations.

To verify that we could isolate cells by flow sorting on the basis of these differences in BiFC signal we transfected bait PCNA_CTV cells with the empty vector or one of the 2 NTV_Fen1 prey plasmids, and analyzed the cells on a MoFlo fluorescence cytometer after 24 hours (**Fig. 2B**). Untransfected cells show only endogenous levels of yellow auto-fluorescence (none are brighter than a threshold set at 108 units). Transfection with empty vector or NTV_Fen1 PIP* constructs do result in low amounts of yellow fluorescence: 3.4% and 4.7% cells, respectively. This represents the cumulative, spontaneous refolding of Venus in the absence of targeted association between fusion partners. However, transfection with NTV_Fen1 WT, which is able to interact with PCNA and mediate specific BiFC resulted in significantly increased yellow fluorescence, with 9.9% cells demonstrating bright yellow fluorescence. Thus the BiFC methodology can be combined with cell sorting to enrich cells with specific BiFC signal.

**Figure 3.**
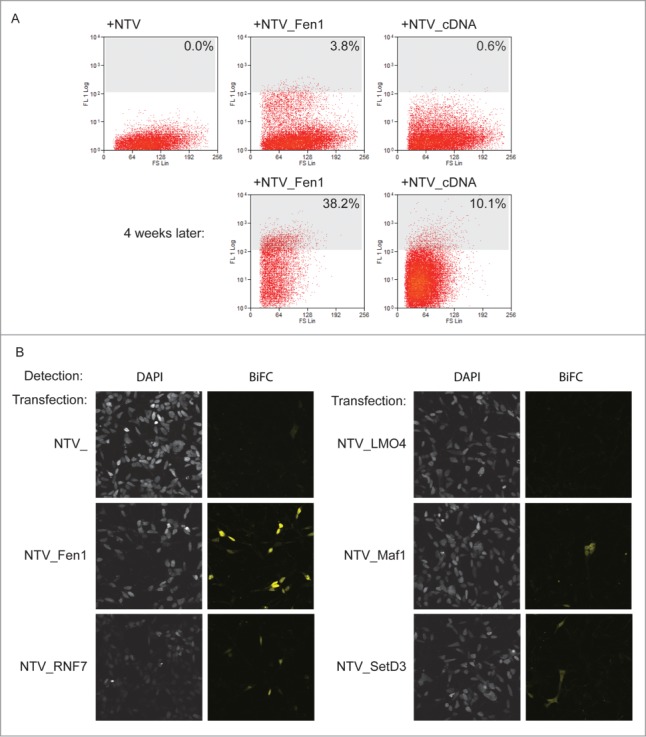
A library screen to identify PCNA interacting proteins. (**A**) The screen in practice. Top panels: FACS analysis of yellow fluorescence of HEK293 cells stably expressing PCNA_CTV 24 hours after transfection as indicated. The cells with a fluorescence level greater than 108 units (% indicated) were collected and returned to culture with selection to maintain the prey plasmids. Four weeks later the fluorescence profiles were as indicated (lower panels). (**B**) Validation of candidate PCNA interacting proteins from the screen. Constructs expressing the full length cloned cDNAs indicated were transfected into HEK293 cells stably expressing PCNA_CTV and BiFC signal was detected in the yellow channel using confocal microscopy.

In order to use this method to characterize the PCNA interactome we converted a human cDNA library into a prey library using Gateway™ mediated recombination. This library was transfected into 5×10^6^ bait PCNA_CTV cells, and cells were analyzed on a MoFlo cytometer for yellow fluorescence after 24 hours. At this time cells with an intensity of greater than 108 were taken as positive for interaction, these comprised 3.8% of the total NTV_Fen1 transfected cells and 0.6% of the cells transfected with the library (**Fig. 3A**). In total 13000 BiFC positive cells were isolated. These were returned to culture. 65000 unsorted but library transfected cells were also returned to culture to act as a control. After 24 hours hygromycin was added to select for cells that had received prey plasmids. The cells were grown under selection for 4 weeks. After this time FACS analysis confirmed that a substantial proportion of the sorted cells were still exhibiting BiFC yellow fluorescence: 38.2% in the control NTV_Fen1-transfected and 10.1% in the cDNA library-transfected population (**Fig. 3A**). After further expansion, DNA was extracted from the library-transfected, sorted population and the unsorted control. These DNA preparations were used as templates for PCR- mediated amplification of the library sequences present using plasmid-specific probes. 500ng of these PCR products were used to generate a 454 sequencing library, which was analyzed in multiplex format on the FLX junior 454 platform. A total of 66694 and 32135 reads was obtained for sorted and control libraries respectively. After trimming of vector sequences and removal of genomic contaminants these reads aligned to 501 and 504 unique cDNAs in the sorted and control screens.

This depth of sequencing enabled identification of the most abundant cDNAs in the starting library, which are most likely to contribute false positive results in the screen. To take this bias into account when analyzing the data we looked specifically at those cDNAs which were rare or not present in the unsorted control dataset, but which were abundant in the sorted screen. To do this in an unbiased way we ranked the cDNAs identified in each data set in order of their abundance. We then calculated the change in rank for each between the library control and the screen enriched populations. A positive change in rank demonstrates a relative enrichment by the screen procedure, which is likely to be the result of true positive interaction. Indeed, using this approach the known PCNA interactors PolD2 and Fen1, were identified by the screen. All cDNAs with a change in rank of greater than 50 places are provided in supplementary **Table 1**. We performed a gene ontology (GO) based analysis using the DAVID algorithm^46^ to determine whether any particular biological pathways or functions were over-represented in the screen. We found however that the gene groups represented in the screen hits corresponded to ribosomal proteins, followed by proteins of oxidative metabolism, and actin and myosin related groups. These pathways were also over-represented in the library control dataset. We therefore concluded that GO analysis was of limited use in this case due to the biased nature of the input material. To validate the screen, we selected 4 cDNAs that were enriched in this PCNA interaction screen, but not previously reported to interact with PCNA, for further study: those encoding LMO4, Maf1, RNF7 and SetD3. These candidates were manually selected because database searches suggested that they are predicted to be nuclear proteins with potentially interesting roles not limited to skeletal muscle and additionally, their size should permit expression in *E. coli*, facilitating initial study.

To confirm that the presence of these cDNAs in the sorted library is a result of a PCNA interaction we cloned the full length cDNAs of LMO4, MAF1, RNF7 and SETD3 into the BiFC prey vector and transfected them independently into bait cells. These were analyzed for yellow fluorescence on a confocal microscope (**Fig. 3B**). MAF1, RNF7 and SETD3 constructs all generated yellow fluorescence resulting from BiFC, showing that these proteins can associate with PCNA. On the other hand, expression of a full-length LMO4 prey construct did not generate detectable BiFC, suggesting that LMO4 is a likely false positive.

**Figure 4.**
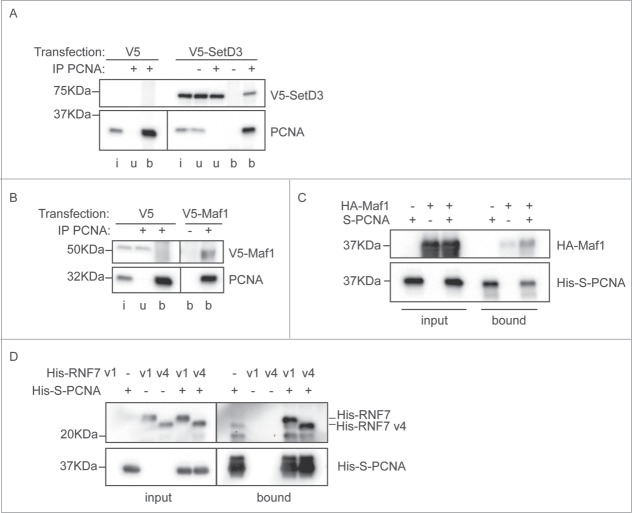
Interaction between PCNA and the newly identified partners. (**A**) SetD3 interacts with PCNA. Soluble nuclease-treated protein extracts were made from MRC5 cells transfected as indicated. Immunoprecipitations were performed using an anti-PCNA polyclonal antibody (+) or a non-specific control IgG (−) and input material (i), unbound material (u) and immunoprecipitated material (b) was analyzed by western blotting using anti-V5 and anti-PCNA primary antibodies. (**B**) Maf1 interacts with PCNA. Immunoprecipitations were performed and analyzed as in A), using extracts from cells transfected with V5-Maf1 or controls as indicated. (**C**) Recombinant Maf1 interacts with PCNA. Recombinant His-HA-Maf1 and His-S-PCNA were individually expressed in *E. coli*. Soluble protein extracts were produced and mixed together or with control extracts (from cells expressing empty vectors or His-HA-GST) as indicated. His-S-PCNA was affinity purified on S-resin and the bound, and input, fractions analyzed by western blotting using anti-PCNA or anti-HA antibodies. (**D**) Recombinant RNF7 interacts with PCNA. Recombinant His-tagged RNF7 variants 1 (v1) or 4 (v4) and His-S-PCNA were individually expressed in *E. coli*. Extracts were produced and mixed together as in C). Proteins associated with S-resin were analyzed by western blotting using anti-His antibodies. In all parts the migration positions of protein molecular weight markers are indicated.

To independently validate the interaction between these proteins and PCNA we performed immunoprecipitations and *in vitro* interaction analyses. A V5-tagged version of SetD3 was transiently transfected into MRC5 cells, from which extracts were made. PCNA was immunoprecipitated from these cell extracts, and precipitates were probed for the presence of co-precipitated V5-SetD3 (**Fig. 4A**). SetD3 specifically co-precipitated with PCNA, indicating that the identified interaction is physiological. Similarly, we expressed V5-tagged Maf1 in human cells by transient transfection. Immunoprecipitation of PCNA using a polyclonal antibody, specifically co-precipitated Maf1 (**Fig. 4B**). This immunoprecipitation only co-purified a small amount of the Maf1 protein, so to further verify the interaction with PCNA we additionally tested it *in vitro*. Recombinant Maf1 protein was expressed in *E. coli*. Soluble *E. coli* extracts containing HA-tagged Maf1 were mixed with *E. coli* extracts containing S-tagged recombinant PCNA, or controls, and proteins were isolated on S-resin. Resin-associated proteins were analyzed by immunoblotting (**Fig. 4C**). This demonstrated that recombinant Maf1 can directly associate with PCNA, validating our immunoprecipitation and BiFC findings. The RNF7 gene is expressed in several isoforms and the sequencing reads from the screen did not enable us to distinguish whether isoform 1 or isoform 4 was able to interact with PCNA. We thus cloned the open reading frames for both these proteins and expressed them in *E. coli*. *In vitro* pull down experiments, as described above for Maf1, show that both RNF7v1 and RNF7v4 specifically associate with PCNA (**Fig. 4D**). Overexpression of either isoform of V5-strep-RNF7 reduced cell viability in MRC5 cells (data not shown) precluding investigation of this interaction by co-immunoprecipitation. Collectively, these results validate the BiFC screening protocol and highlight 3 novel PCNA interacting proteins that may have important roles in DNA replication, repair, or other cellular pathways involving PCNA.

During S phase of the cell cycle PCNA becomes concentrated in replication factories in the nucleus, which can be visualised as focal nuclear substructures using fluorescence microscopy.^40,41,47^ We assessed the sub-nuclear localization of Maf1, SetD3 and RNF7 by transfecting MRC5 cells with constructs expressing GFP-tagged versions targeted to the nucleus with a nuclear localization signal (NLS). Co-transfection of RFP-tagged PCNA was used to indicate the positions of replication factories in S phase cells. We could not detect localized enrichment of Maf1, SetD3 or RNF7 at replication factories (**Fig. S1**). Furthermore, while PCNA is retained at replication foci after extraction of nuclear soluble proteins with detergent prior to fixation, most signal from Maf1, SetD3 and RNF7 is removed by even very limited detergent treatment (**Fig. S1**). This suggests that if these proteins are involved in DNA replication, this role does not require enrichment or tight retention of their activity at replication sites. PCNA also has a role during the DNA synthesis phase of nucleotide excision repair,^48^ and it is specifically recruited to damaged DNA sites.^49^ We used a localized UV-irradiation procedure to assess whether Maf1, SetD3 or RNF7 are enriched at sites of UV induced damage.^50^ Immunofluorescence using an anti-XPA antibody was used to mark the position of ongoing DNA repair. No increased concentration of Maf1, SetD3 or RNF7 proteins was detected at repair sites (**Fig. S2**). While detergent resistant recruitment of PCNA was readily detected at sites of localized irradiation, extraction with triton prior to fixation removed all the Maf1, SetD3 and RNF7 signal (**Fig. S2**). This suggests that, as for replication, if these proteins are involved in nucleotide excision repair, this role does not require enrichment or tight retention of their activity at repair sites. It is certainly possible that these proteins might have active roles at replication or repair sites that are not detected by these approaches, or alternatively that the PCNA interactions are used during other cellular processes that we have not yet analyzed.

## Discussion

As a key player regulating the events of chromosomal replication PCNA is fundamental to genome and epigenome stability. Recently the first pathogenic mutation in PCNA in humans was reported, and was shown to cause disease by its inability to properly interact with a subset of protein partners.^51,52^ This additionally raises the possibility that the loss or mutation of PCNA-interacting proteins could similarly contribute to human disease. Thus, understanding the extent, mechanism and regulation of protein traffic on PCNA is of great importance. Although PCNA was identified as critical for DNA replication and repair almost 20 years ago,^53^ there still has not been a systematic investigation of the PCNA-interactome, although the need for such a study has been highlighted.^4,35^ Instead there have been several independent studies using a variety of methodologies generating as many as 238 reported PCNA interaction partners to date (http://thebiogrid.org/111142). Here, we sought to design a strategy capable of detecting all potential PCNA interactions in human cells. To do this we utilised the BiFC methodology. We reasoned that this was eminently suitable for our question because: a) It places the interactions in a human cell system that should recapitulate any post-translational modification events that may be necessary for interaction. b) Our system targets the expressed reporters to the nucleus, thus enabling discovery of interactions that occur within a relevant intracellular environment. c) The refolding of the BiFC split-Venus construct has been reported to stabilize interactions between bait and prey constructs.^54^ Although this may result in higher false positive rates (as we did observe) this phenomenon should enhance the detection of weak or transient interaction events that might be missed by other methods. In addition this screen provides real-time readout of interaction and is high throughput in format. These characteristics means that this format can be readily developed in the future to study dynamic changes in the PCNA interactome such as might occur upon drug treatment or other cellular stresses. Here we utilised HEK293 cells as a readily transfectable human cell line, but the method should be applicable to other cell types.

The PCNA interaction partners that we identified here have not previously been reported to have a role in DNA replication. SetD3 is a conserved histone H3-methyltransferase.^55,56^ It is abundantly expressed in muscle and promotes muscle-differentiation by regulating the transcription of muscle-related genes. In our experiments the BiFC signal from the PCNA-SetD3 interaction is found both in cytoplasm and nucleus. Consistent with this, the endogenous protein has been reported previously to localize to both compartments in HeLa cells.^57^ The exogenous overexpression of *Danio rerio* SetD3 in either mouse or human cell lines led to decreased cell viability and increased apoptosis.^56^ In contrast, we have generated cells expressing human SetD3 with either V5- or HA_NTV- epitope tags without observed perturbations to cell proliferation or viability (data not shown). The *D. rerio* and human SetD3 proteins share 78% identity, differing mostly in their C-termini, which contain a putative substrate binding domain. It therefore seems possible that species-specific binding events at the C-terminus of SetD3 mediate this toxicity, and as such this domain warrants further investigation.

The *MAF1* gene is conserved from yeast to humans.^58^ In *S. cerevisiae* it is a negative regulator of RNA polymerase III in response to lack of nutrients and growth factors, and replication stress,^59,60^ and in humans it can regulate all 3 RNA polymerases.^61^ Maf1 has been suggested to have a tumor suppressor function: it represses oncogenesis, reducing anchorage-independent growth and tumor formation in mice^62^ and human cells,^61^ and in *Drosophila melanogaster*, dMaf1 depletion leads to an increase in growth rate and body size.^63^ Although both our *in vitro* and in cell experiments have shown a weak interaction between human Maf1 and PCNA, we do not yet know the biological function of this association.

The *RNF7* gene (alternatively known as *RBX2*) is also highly conserved during evolution (it is the homolog of *S. cerevisiae* Roc2) and the protein is present in both the cytoplasm and the nucleus in human cells.^64^ It was first identified as a redox-inducible and apoptosis-protective antioxidant protein that decreases the production of ROS.^64,65^ It is overexpressed in various cancers, including lung, colon, stomach and liver cancer.^66,67^ More recently it was shown to be a component of specific Cullin-RING E3 ubiquitin ligases, in which it binds Cullin-1 or Cullin-5 to promote the ubiquitination and subsequent proteasomal degradation of substrates.^68,69^ It is of note that one of PCNA's cellular roles is to target PIP degron-containing substrates to the Cul4/DDB1 ubiquitin ligase.^22-24^ This complex contains RNF75 (RBX1), which has significant homology to RNF7. Although no direct interaction has been reported between PCNA and RNF75, given our results it will be of interest to ascertain whether there is a direct physical binding of PCNA to RNF75 that could be important for this degradation function. Alternatively PCNA might contribute, via the RNF7-interaction, to the proteasomal degradation of other proteins that are not targets of the Cul4/DDB1 ligase. In *S. cerevisiae*, a yeast 2-hybrid assay shows no interaction between Roc2 and PCNA,^70^ suggesting that the role of this interaction may be specific to higher eukaryotes.

We note that none of the interaction partners here identified contain canonical PCNA interaction motifs. This suggests there are additional modes of PCNA interaction, not previously detected by the predominantly candidate-based approaches for identification of PCNA interactors that have been utilised to date. The three novel interaction partners reported here also do not appear to be enriched at DNA replication factories nor nucleotide excision repair sites in human cells. There are many reasons why this might be the case. It is now clear that PCNA interactions can be utilised to initiate protein degradation, thus Maf1, SetD3 or RNF7 might be destroyed following PCNA binding. It is also possible that the interactions with these factors are not utilised at the DNA replication forks, but during other cellular functions in which PCNA plays important roles. The screen reported here used a cDNA library derived from skeletal muscle. As a non-proliferating, non UV exposed tissue it is possible that the screen will actually be biased against detection of proteins involved in DNA replication and nucleotide excision repair. PCNA is crucial for other processes of DNA repair: base excision repair and probably homologous recombination,^71-74^ and it is also possibly involved in transcription and signaling,^4^ thus the novel interactions identified here may well be required for other important biological pathways in which PCNA is implicated. It is clear that even subtle perturbation to PCNA's partner profile can cause unexpected cellular phenotypes and have dramatic consequences for human health.^52^ The identification of these novel PCNA partners provides a new avenue for understanding the multifaceted and complex roles of this fascinating protein.

## Materials and Methods

### Cell lines and transfection

MRC5 (SV40-transformed) and HEK293 and derived lines were grown in DMEM glutamax (Invitrogen) with 10% FBS, supplemented with penicillin and streptomycin at 37°C; 5% CO_2_. Cells were transfected using PEI or FugeneHD. Selection was with 750 μg/ml G418 or 100 μg/ml hygromycin where used.

### Plasmids

For BiFC experiments, human cDNA encoding PCNA was cloned into the bait vector, which was derived from pcDNA3.1 (Invitrogen) engineered to produce the C-terminal portion of Venus fluorescent protein (amino acids 159–230), a nuclear localization signal (PKKKRK), the FLAG epitope (DYKDDDDK) and a flexible hydrophilic linker sequence (GEGQGQGQGPGRGYAYRS). cDNAs encoding human Fen1, RNF7v1 and v4, Maf1 and SetD3 were cloned into an engineered pDEST expression prey vector derived from pCEP4 (Invitrogen) containing the HA epitope (YPYDVPDYA) the N-terminal portion of Venus (amino acids 1–158) and an NLS and linker as above and attR sites for recombination; using the Gateway system (Invitrogen). For DNA replication factory and DNA repair site analyses, human cDNAs encoding Maf1, RNF7v1 and v4 and SetD3 were transferred into an engineered pDEST expression vector with an NLS, GFP-tag and the same flexible linker sequence as above, human cDNA encoding Fen1 was cloned in pHAGE–GFP^75^ and PCNA with an N-terminal NLS-mRFP tag was also used.^76^ For bacterial recombinant protein expression the open reading frames were transferred into engineered Gateway pDEST expression vectors derived from pET30a (His- and S-tag), or pET16b (His- or His- and HA-tags) (Novagen). For immunoprecipitations the open reading frames were transferred into an engineered Gateway pDEST expression vectors derived from pcDNA3.1/NV5 DEST (Invitrogen). Plasmids and sequences are available on request.

### cDNA library

The cDNA library used was derived from skeletal muscle (male, 24 years) with an average insert size of 1.6 kb in the pCMV•SPORT6 vector (Invitrogen catalog number 11327–012). The library cDNAs were exchanged into the pDONRzeo vector (Invitrogen) using a BP reaction according to the manufacturers protocols (Invitrogen). An LR reaction was then used to transfer the cDNA library into the engineered prey pDEST vector based on pCEP4 as above.

### Fluorescence activated cell sorting

Cells were analyzed and sorted on a MoFlo cytometer (Beckman Coulter) in FACS sort buffer: HBSS supplemented with 25 mM HEPES and 5 mM EDTA. A 100μm nozzle was used at 30psi. Venus fluorescence was detected with a 100mW, 514 laser line with a 546 nm filter. After sorting cells were returned to conditioned medium to aid recovery.

### DNA preparation and PCR

DNA was prepared from sorted cells and unsorted controls using the GeneJet DNA purification kit (Thermo Scientific) according to the manufacturer's instructions. This was used as a template to amplify library cDNA inserts by PCR using Phusion DNA polymerase (New England Biolabs) and pCEP4-specific primers (CATTATGCCCAGTACATGACCTT and GCAATAGCATCACAAATTTCACA) according to manufacturer's protocols.

### 454 sequencing

500 ng PCR product from the above reactions was used submitted for 454 Alicon sequencing using the Roche FLX Junior sequencer in the Department of Biochemistry, University of Cambridge. Raw read data was trimmed using Flexbar to remove vector and adapter sequences and then blastn was used to query the human nucleotide genomic and transcript databases for sequence matches. Genomic contaminants carried over from the PCR reactions were removed manually, as were mitochondrial genes.

### Immunofluorescence and EdU localization

Cells growing on glass coverslips were either fixed directly in 2% formaldehyde in PBS for 20 minutes at room temperature, or treated with 0.1% or 0.2% Triton X-100 in CSK (10 mM Pipes, pH 7.0; 300 mM sucrose; 100 mM NaCl; 3 mM MgCl_2_; 1× complete protease inhibitor cocktail (Roche)) for removal of soluble nuclear proteins prior to fixation. Where used, 12μM EdU was added to culture medium for 10 minutes prior to fixation. EdU was visualised using the Click-iT® EdU Alexa Fluor® 647 Imaging Kit (Invitrogen) according to the manufacturer's instructions. Immunofluorescent detection of bait and prey proteins was using anti-FLAG M2 antibody (Sigma) or anti-HA monoclonal antibody 12CA5 (Abcam). XPA was detected using anti-XPA monoclonal antibody (abcam). After permeabilisation of cells (0.5% Triton X-100 in PBS for 5 minutes), blocking was for in 3% BSA (Sigma) solution in phosphate buffered saline (Gibco) containing 0.1% Tween-20 (Sigma). Primary antibodies were incubated on cells at a 1/1000 dilution in blocking buffer for 2 hours at room temperature. After washing 3 times in blocking buffer secondary antibody goat-anti-mouse-Alexa Fluor 488 or 633 (Molecular Probes) was used at a dilution of 1/1000 in blocking buffer for 1 hour. After three further washes in blocking buffer coverslips were mounted in Aqua-Poly mount with DAPI. Immunofluorescent signal was visualised on a Leica TCS confocal using a 63× objective with oil immersion. The following excitation (Ex.) and emission (Em.) settings were used: DAPI: Ex. 405 nm; Em. 415–518 nm. GFP/Alexa Fluor 488: Ex. 488 nm; Em. 500–600 nm. Venus (BiFC): Ex. 514 nm; Em. 524–618 nm. RFP/Alexa Fluor 555 : Ex. 543nm; Em. 561–615 nm. Alexa Fluor 633/647: Ex. 633 nm; Em. 645–769 nm.

### Recombinant protein production

His-S or His-HA tagged fusion proteins were produced in BL21-CodonPlus (DE3)-RIPL competent cells (Agilent) at 30°C for 4h after addition of 1mM IPTG. Cells were collected by centrifugation and lysed in cold PBS with 1mM PMSF and 0.1% Triton X-100, using a Diagenode Bioruptor (8 cycles of 30s on High). Soluble fractions were kept at −80°C.

For PCNA *in vitro* interaction, lysates were mixed and then incubated with S-agarose beads (Novagen) at 4°C overnight with agitation. Beads were washed in cold PBS with 0.1% Triton X-100 and then resuspended in Laemmli SDS-PAGE buffer. Negative controls were performed using lysates from cells expressing only His-S or His-HA-GST.

### Cell extracts

HEK or MRC5 cells were washed in PBS with 1mM iodoacetamide then incubated 30min on ice in extract buffer (10% glycerol, 0.01% Igepal, 40 mM NaCl, 50 mM Tris pH7.5, 2 mM MgCl_2_, 1U/ml Benzonase (Novagen) and 1 × complete protease inhibitors (Roche)). The NaCl concentration was adjusted to 150mM and samples were incubated a further 10 minutes on ice. Cell lysates were centrifuged at 17000 × g, for 15 minutes at 4°C. Protein concentration was determined by Bradford assay (Sigma) and then adjusted to equalise by the addition of extract buffer.

### Immunoprecipitation

For analysis of PCNA_CTV bait binding to endogenous PCNA Flag pull-downs were performed by incubating extracts from HEK 293 PCNA_CTV stable cell lines, or controls, with Anti-FLAG M2 Magnetic Bead (Sigma) for 2h at 4°C. After washing, beads were resuspended in Laemmli SDS-PAGE buffer and proteins analyzed by western blotting using an anti-PCNA monoclonal antibody PC10 (Abcam). For analysis of PCNA interactions, extracts from MRC5 cells transfected with V5-tagged prey vectors were made as above. Before immunoprecipitation, a buffer exchange was performed using Zeba spin desalting column (Pierce) into PCNA-binding buffer (10% glycerol, 0.01% Igepal, 25 mM NaCl, 25mM Tris pH7.5, 1× complete protease inhibitors (Roche); conditions shown to be conducive to the detection of PCNA containing complexes.^77^ Immunoprecipitation was performed by incubating cell extracts with rabbit polyclonal PCNA antibody (Abcam) or control rabbit IgG (Dako) for 2h followed by overnight incubation with Dynabeads protein A/G (Invitrogen) at 4°C. After washing, beads were resuspended in Laemmli SDS-PAGE buffer and proteins analyzed by western blotting using anti-HA monoclonal (Abcam) or PCNA monoclonal PC10 (Abcam).

### Western-blot analysis

Proteins were resolved by SDS-PAGE, transferred to a PVDF membrane (Whatman), and probed with the following antibodies: mouse anti-PCNA (ab29), rabbit anti-V5 (ab9116) and mouse anti-HA (ab16918) from Abcam. Membranes were then incubated with the appropriate HRP-conjugated secondary antibodies and detected by chemiluminescence using a ChemiDoc MP System (Bio-Rad) and Clarity or DURA detection reagents (Bio-Rad/Pierce). For RNF7 experiments, an additional 2mM DTT were added to the sample buffer in order to reduce samples and obtain monomers.^65^

### Localized UV irradiation

MRC5 were transfected at least 24h prior to irradiation. Irradiation was performed as previously described.^49^ Briefly, cells grown on coverslips to around 80% confluency were washed in PBS. Excess PBS was removed and isopore membrane filters with 5 µm pores (Millipore) were placed on top of cells before irradiation with UVC (254 nm) at 100J/m^2^. After filter removal, cells were put back in medium and allowed to recover for 30 minutes in the incubator prior to further treatment or fixation.
