# Supplementary material for: A fluorescent bimolecular complementation screen reveals MAF1, RNF7 and SETD3 as PCNA-associated proteins in human cells
Source: Cell Cycle. 2015 Jun 1;14(15):2509–19. doi: 10.1080/15384101.2015.1053667 (PMC4613188; doi:10.1080/15384101.2015.1053667)
Supplement: 1053667_supplemental_files.zip [file kccy-14-15-1053667-s001.zip › 1053667 supplemental files/Figure S1 version 3.pdf]

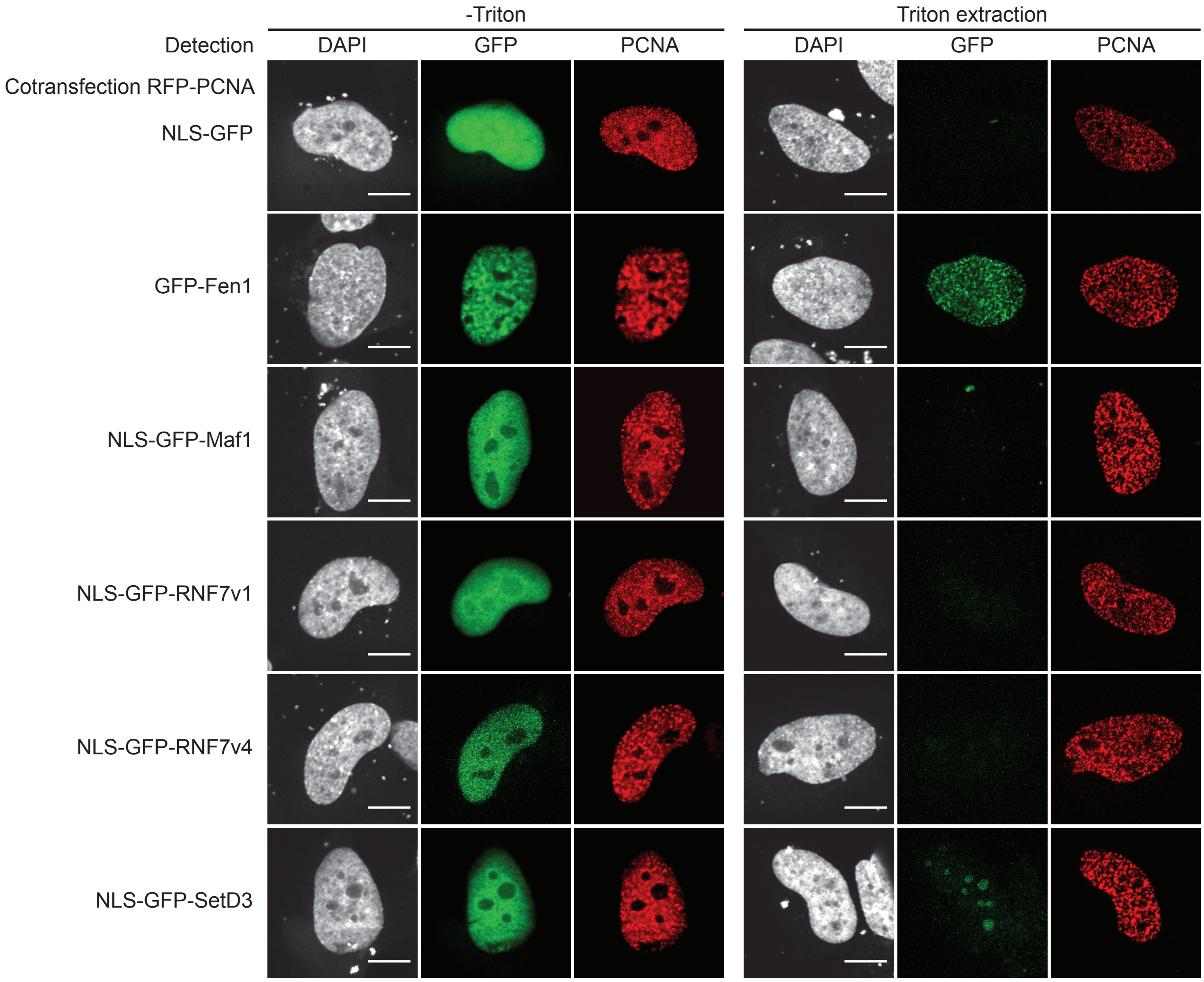

Supplemental figure 1. Analysis of DNA replication factories.
MRC5 cells were transfected as indicated and analysed by confocal microscopy directly or after extraction of soluble nuclear proteins with 0.2% triton for 1 minute on ice. RFP-PCNA foci mark the replication factories. No specific enrichment of Maf1, RNF7 or SetD3 is seen at these sites. Scale bar = 10μm.
