# Supplementary material for: A fluorescent bimolecular complementation screen reveals MAF1, RNF7 and SETD3 as PCNA-associated proteins in human cells
Source: Cell Cycle. 2015 Jun 1;14(15):2509–19. doi: 10.1080/15384101.2015.1053667 (PMC4613188; doi:10.1080/15384101.2015.1053667)
Supplement: 1053667_supplemental_files.zip [file kccy-14-15-1053667-s001.zip › 1053667 supplemental files/Figure S2 version 4.pdf]

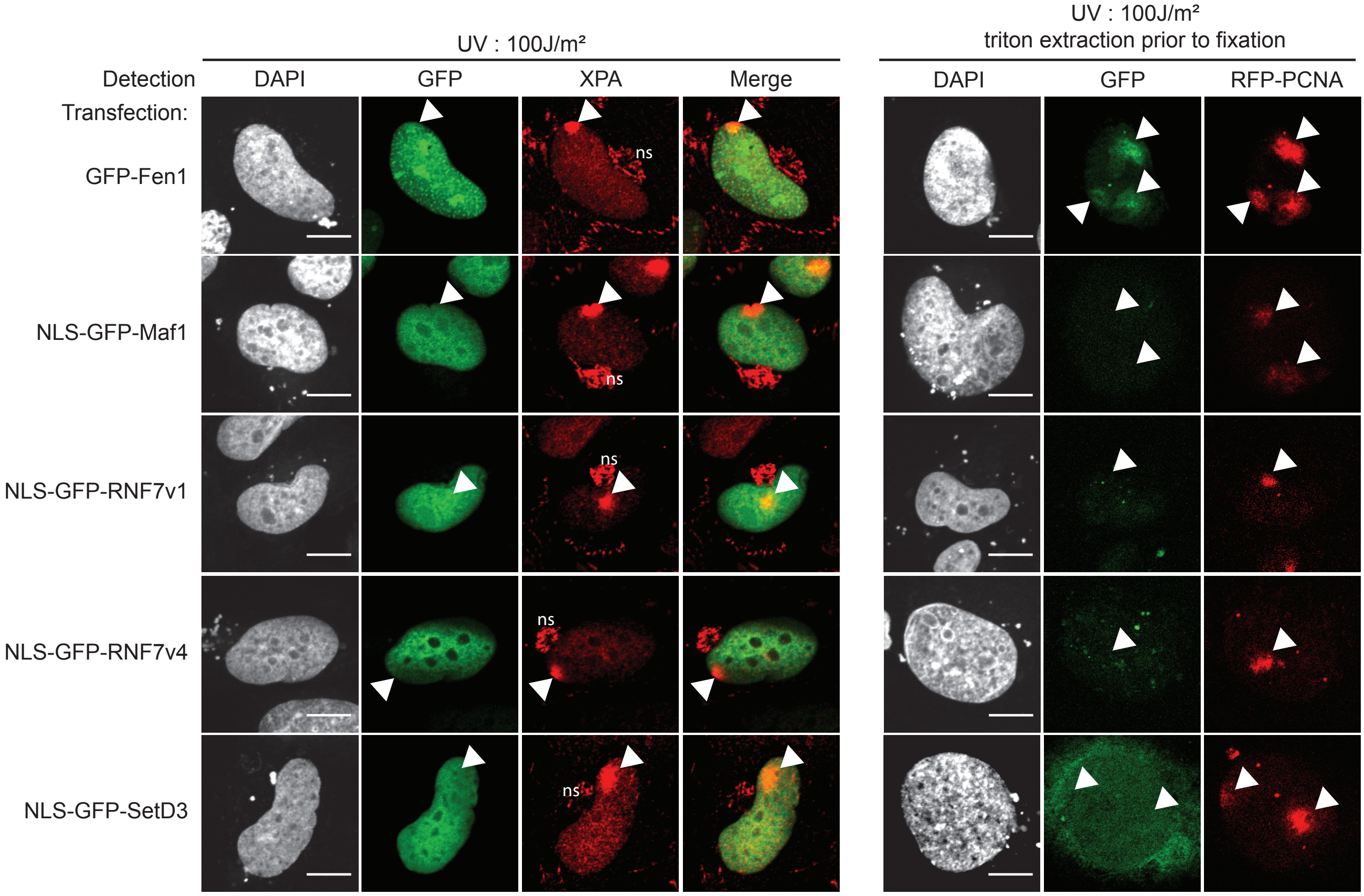

Supplemental Figure 2. Analysis of DNA repair sites.

MRC5 cells were transfected as indicated then subjected to localised UV irradiation (100J/m^2 through an Isopore membrane filter with 5μm pores), allowed a 30 minute post-irradiation recovery period and then analysed by confocal microscopy directly or after extraction of soluble nuclear proteins with 0.2% triton for 1 minute on ice. Indirect immunofluorescence using an anti-XPA antibody, or RFP-PCNA was used to visualise the repair sites (arrowheads). The anti-XPA antibody used generates non-specific cytoplasmic staining adjacent to the nucleus (ns) and also at the cell periphery, in addition to the specific nuclear signal. Mock irradiated samples showed no characteristic nuclear repair patches. No specific enrichment of Maf1, RNF7 or SetD3 is seen at the repair sites. Scale bar = 10μm.
